# Amaranth Seeds as Lactic Acid Bacteria Fermentation Substrates: Prospects for Metabiotic Foods and Functional Ingredients

**DOI:** 10.3390/foods15152645

**Published:** 2026-07-28

**Authors:** Aida Mihaela Vasile, Marina Pihurov (Procop), Mihaela Cotârleț, Gabriela Elena Bahrim

**Affiliations:** 1 Faculty of Food Science and Engineering, Dunărea de Jos University of Galați, 111 Domnească Street, 800201 Galați, Romania; aida.vasile@ugal.ro (A.M.V.); gabriela.bahrim@ugal.ro (G.E.B.); 2 Institute of Microbiology and Biotechnology, Technical University of Moldova, 3/3 Academiei Street, MD-2028 Chisinau, Moldova

**Keywords:** amaranth, lactic acid bacteria, functional foods, bioactive compounds, antinutrient reduction, polyphenol activation, germination, metabiotic ingredients

## Abstract

Amaranth, a nutritionally dense pseudocereal, is an exceptional substrate for producing functional and metabiotic food products via lactic acid fermentation. This review provides a comprehensive analysis of both ungerminated and germinated amaranth seeds as fermentation matrices, assigning their nutritional profiles, bioactive constituents, and compatibility with microbial transformation. Central to this discussion are the synergistic interactions between amaranth’s native compounds and lactic acid bacteria (LAB), with an emphasis on four key bioconversion mechanisms: protein hydrolysis, polyphenol activation, antinutrient reduction, and the biosynthesis of functional and metabiotic metabolites. Essential fermentation parameters, including substrate preparation, inoculation strategies, pH, temperature, and fermentation time, are systematically reviewed to guide optimization of bioactive compound yields. Post-fermentation processing approaches, such as freeze-drying, controlled drying, and product standardization, are assessed for their capacity to preserve product stability and ensure consistent functional performance. The review further explores the incorporation of fermented amaranth into diverse food systems, such as gluten-free bakery goods, functional beverages, and nutraceutical formulations, as well as its application in animal nutrition, where it supports improved digestibility and gut microbiome health. The review concludes by mapping current challenges, unresolved knowledge gaps, and priority research directions, positioning fermented amaranth systems as versatile, science-backed platforms for developing next-generation functional and metabiotic ingredients.

## 1. Introduction

Pseudocereals are dicotyledonous plants that differ from traditional cereals in structure and function. They are underutilized, gluten-free crops high in protein and rich in nutrients and saponins, with valuable applications in the agro-pharma and agro-industrial sectors. Considered 21st century crops with complete nutritional profiles, they nonetheless contain mineral absorption inhibitors such as phytates and other antinutritional compounds [1], while the rising global demand for gluten-free flours, driven by an increasing number of celiac patients, continues to highlight their importance; ecologically, they enhance and diversify natural resources, and despite being eco-friendly, functional, affordable, and resilient to adverse conditions, they still present certain growth challenges [2]. Among pseudocereals, amaranth stands out as one of the oldest food crops in human history, cultivated as far back as 6700 BCE in the Americas; a warm-season, self-pollinating annual, it reached its peak as a staple food during the Aztec era in Mexico in the 1400s and has since expanded over the past two centuries to regions including Mexico, Central America, India, Nepal, China, and East Africa, with US research beginning in the 1970s, though optimal production standards and uniform varieties remain under development [3].

At the global level, grain amaranth (*Amaranthus* spp.) is still considered a minor and underutilised crop. In this sense, global production statistics for this species are not consistently reported by the FAO [4]. China is one of the top exporters of amaranth, while India leads the world market in both production and market share. Germany is the biggest consumer market in Europe. As amaranth’s nutritional and functional qualities become more widely known, demand is steadily rising in the UK, the Netherlands, Belgium, Sweden, France, and Romania [5].

Amaranth is a nutrient-rich pseudocereal high in protein, minerals, and bioactive compounds such as phenolics and flavonoids. Its agronomic versatility and traditional uses underscore its strong potential as a functional food ingredient. Its seeds are enclosed within a utricle, classified as dehiscent, semi-dehiscent, or indehiscent, measuring 0.9–1.7 mm in diameter and ranging in color from cream to black; these tiny, pale, lens-shaped seeds are borne on seed heads 30–112 cm wide and 13–61 cm tall [6].

Despite its widespread use, amaranth remains relatively understudied from an agronomic perspective. Grain yields are highly variable across sites and genotypes, and the role of plant density in maximizing yield, while recognized as critical, is still insufficiently documented. Contemporary research efforts have concentrated on adapting amaranth to mechanized harvesting systems—an objective that entails reducing plant height, minimizing seed shattering, and optimizing flowering timing, maturity uniformity, and overall grain quality. Hybrid development presents an additional layer of complexity owing to amaranth’s predominantly self-pollinating nature and intricate floral architecture; genetic male sterility has been proposed as a viable pathway forward. A fundamental agronomic constraint lies in the photoperiod sensitivity of many amaranth species, which complicates genotype-to-environment matching across diverse climatic conditions [7]. Beyond agronomic limitations, the presence of antinutritional compounds constitutes a significant barrier to broader adoption of amaranth in the food industry. As with other raw cereals, unprocessed amaranth harbors a range of toxins and antinutritional factors, including phenolics, saponins, tannins, phytic acid, oxalates, protease inhibitors, nitrates, polyphenols, and phytohemagglutinins. Among these, oxalates and nitrates are of particular concern in fodder applications, while compounds such as oxalic acid, phytates, saponins, and alkaloids impair nutrient bioavailability and, at elevated concentrations, may pose health risks [8]. Various processing techniques have been developed to reduce these compounds; however, each approach entails trade-offs with the nutritional profile. Baking effectively lowers antinutritional content, whereas puffing may compromise amino acid integrity, and roasting reduces protein and starch digestibility. Extrusion diminishes phytic acid, polyphenols, and iron levels while simultaneously increasing soluble fiber content. Soaking and germination reduce phytates and tannins but elevate phenolic concentrations. Fermentation, meanwhile, leads to reductions in both mineral content and antinutrient levels [9,10].

While the existing literature has predominantly focused on the botanical characteristics and physicochemical properties of amaranth as a whole plant, comparatively limited attention has been devoted to the functional potential of its seeds. This review addresses that gap by drawing on current research to examine the underutilized applications of amaranth seeds in germination and fermentation processes, with particular emphasis on fermentation mediated by lactic acid bacteria strains. Furthermore, it explores the integration of the resulting fermented products into food systems, animal feed, dietary supplements, and cosmetic formulations as a strategy for enhancing their nutritional value, bioactivity, and overall functionality.

**Figure 1:**
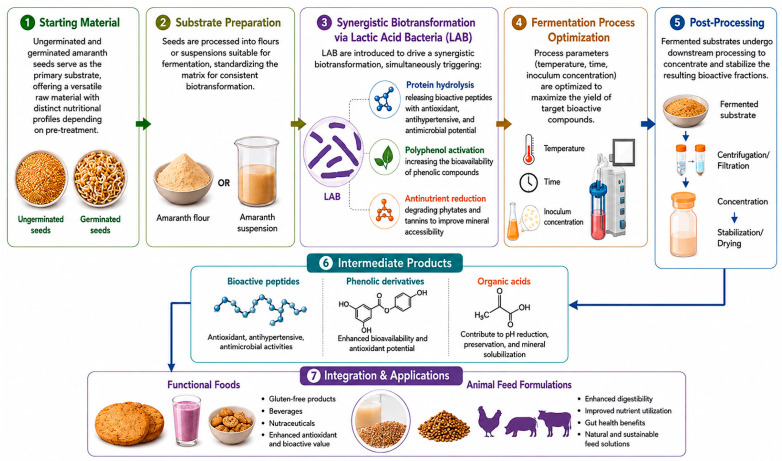
Schematic representation of the enhancement of amaranth functional properties through lactic acid bacteria fermentation (created with Biorender.com).

The schematic representation shown in Figure 1 summarizes the synergistic effects of germination and LAB fermentation on the bioactive potential and functional applications of amaranth.

## 2. Publication Trends, Citation Impact, and Market Statistical Indicators

### Literature Search Strategy

Briefly, the literature search spanned 48 years, from 1977 to 2025, to examine the evolution of *Amaranthus* spp. fermentation research from its inception to the present. The search was conducted in the Web of Science database to maximize the identification of relevant studies, using Boolean operators to combine the terms “amaranth” and “fermentation.” All types of published research materials were included to ensure comprehensive coverage of the subject. A “snowballing” approach was used, with manual review of references from key papers and reviews to avoid missing critical evidence [11]. Titles and abstracts were initially screened for relevance, followed by full-text assessments. Data from the refined Web of Science search were organized in Excel, and visualizations related to graphics and sustainable goals were generated.

**Figure 2:**
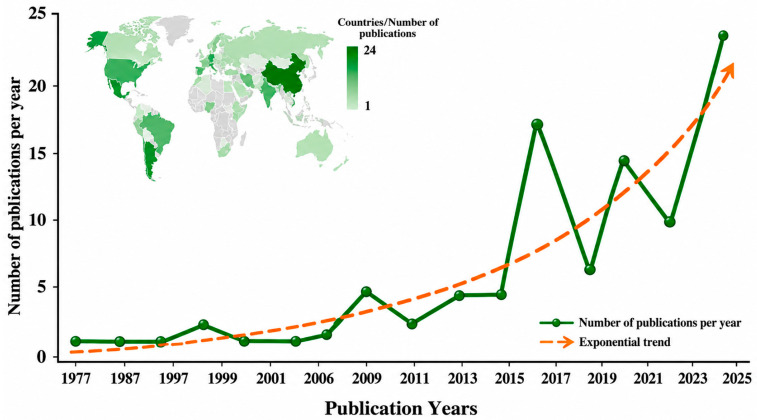
Number of articles published per year on amaranth and fermentation, along with the distribution of publications by country from 1977 to 2025, based on data retrieved from the Web of Science (last accessed 30 November 2025; search conducted using the keywords “amaranth” and “fermentation”) [12].

Research on amaranth and its fermentation has grown substantially over the past decade (Figure 2), a trend driven by key advances across several interconnected fields: the development of sophisticated analytical technologies capable of identifying bioactive, antinutritional, and non-nutritional compounds; the refinement of germination techniques; and progress in genetic seed adaptation and hybridization aimed at improving production efficiency.

Additionally, a search on Web of Science using the keywords “amaranth” and “fermentation” shows 191 publications, revealing their connection to 8 of the 17 United Nations Sustainable Development Goals (SDGs). Specifically, 45.4% relates to goal 3 (Good Health and Well-Being), 24.5% to goal 2 (Zero Hunger), 16.1% to goal 13 (Climate Action), 5.2% to goal 12 (Responsible Consumption and Production), 4.4% to goal 7 (Affordable and Clean Energy), 2.8% to goal 6 (Clean Water and Sanitation), 1.2% to goal 15 (Life on Land), and 0.4% to goal 4 (Quality Education). This link between amaranth fermentation research and these 8 SDGs highlights its broad scientific importance, its potential for sustainability, and its growing global relevance to health, climate resilience, and responsible food production. Among the categories of publication fields in the Web of Science, the largest share of publications was in Food Science and Technology (51.83%), followed by Nutrition and Dietetics (10.99%), Microbiology (9.95%), Agriculture, Dairy, and Animal Science (9.42%), Biotechnology and Applied Microbiology (7.33%), Applied Chemistry (6.80%), Agriculture, Multidisciplinary (4.71%), with the same percentage recorded for Agronomy, and Plant Science (3.67%). Smaller contributions of the overall output were also seen across various engineering fields, as well as in environmental sciences and other branches of chemistry and biochemistry. Overall, amaranth and its fermentation play a vital role and interest in food, nutrition, and microbiology fields, and interest continues to increase.

According to the Amaranth Market Analysis [13], the global amaranth market is projected to reach USD 11.8 billion by 2025, expanding at a compound annual growth rate (CAGR) of 10.3% to attain USD 31.5 billion by 2035. This upward trajectory is largely attributed to the worldwide shift toward clean-label and plant-based dietary patterns, which has amplified demand within the functional foods and nutraceuticals sectors. Conversely, traditional grain producers fail to diversify risk market stagnation. Between 2020 and 2024, growth was primarily fueled by rising consumer interest in ancient grains, gluten-free products, and superfoods. The regulatory landscape governing amaranth markets varies considerably across regions. In the United States, well-established labelling frameworks govern gluten-free and organic designations, while the European Union enforces stringent organic certification standards. China actively funds domestic amaranth production, though import barriers continue to limit external supply chains. Japan’s regulatory environment supports functional foods with permissible health claims, whereas South Korea encourages product innovation subject to mandatory approval processes. India, meanwhile, promotes millet-inclusive farming with comparatively minimal regulatory requirements. Collectively, these market dynamics indicate that health-oriented trends and evolving dietary preferences will continue to drive amaranth market growth, positioning innovation-ready companies to capitalize on opportunities that traditional grain producers may be ill-equipped to capture [13].

## 3. Nutrition Composition of Amaranth

Amaranth is a gluten-free, climate-resilient pseudocereal valued for its high-quality, lysine-rich protein, unsaturated oils, squalene, dietary fiber, tocopherols, tocotrienols, phenolic compounds, and flavonoids. It is rich in minerals and vitamins, offering greater nutritional value than many cereals and several legumes. Its composition varies by variety, cultivar, and growing conditions (Table 1).

**Figure 3:**
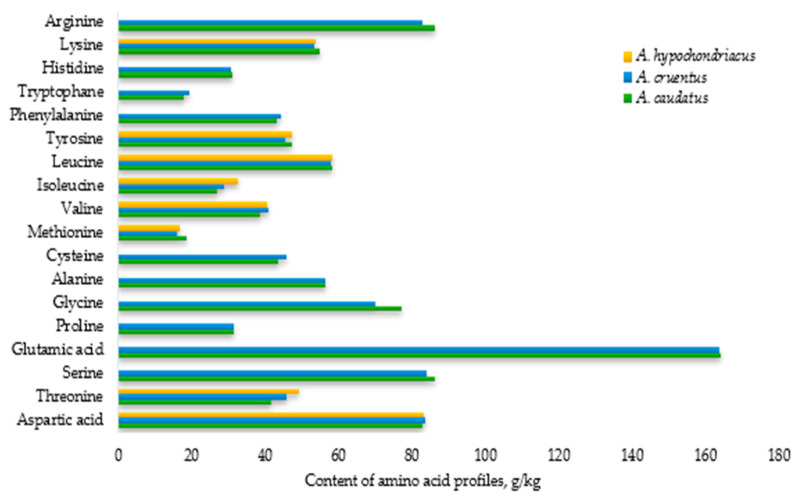
Amino acid composition of amaranth seeds (adapted from [25]).

The amino acid profile of amaranth (Figure 3) contributes significantly to its nutritional and fermentative potential. Essential amino acids such as lysine, leucine, valine, isoleucine, methionine, and threonine support protein synthesis, immune function, and metabolic processes, while glutamic acid, glycine, and alanine participate in energy metabolism and the formation of bioactive compounds. During lactic acid bacterial fermentation, these amino acids act as substrates for microbial growth and contribute to the production of flavor compounds, antioxidants, and health-promoting metabolites, thereby enhancing the functional value of fermented amaranth products [22,23]. A study identified vanillic acid and kaempferol as the predominant phenolic acid and flavonoid, respectively. Oleic acid was the major fatty acid, and unsaturated fatty acids accounted for more than 83% of total fatty acids [24].

## 4. Amaranth Diversity, Germination, and Nutritional Changes

Amaranth exhibits broad genetic diversity across its three cultivated grain species—*Amaranthus cruentus*, *A. hypochondriacus*, and *A. caudatus*—as well as their wild relatives. This diversity is reflected in extensive morphological variation, encompassing seed color (white, cream, brown, black, reddish, or pink), plant height (ranging from a few centimetres to over 2 m), and structure (lenticular to subglobose, with a floury perisperm) [26]. Such variability underscores the importance of carefully selecting appropriate species and ecotypes to optimize germination and fermentation outcomes, as these morphological and genetic traits directly influence seed composition, enzyme activity, and final product quality.

Germination is a cost-effective way to enhance the nutritional value of pseudocereals, legumes, and grains. It is recognized for developing functional foods from amaranth and is a key step in preparing foods like dosa. Although amaranth sprouts are less common than cereal sprouts, their popularity is rising among health-conscious consumers [27]. Germination activates enzymes that break down proteins and carbohydrates and generate bioactive compounds, including phenols, organic acids, peptides, and amino acids. It also alters amaranth’s functional properties by reducing paste viscosity, primarily through amylase-driven starch hydrolysis, which lowers starch molecular weight and swelling capacity, and through disruption of protein disulfide bonds, which weakens gel-forming networks [28]. The most notable changes in chemical composition were observed in flours obtained through germination. Protein content increased substantially by 28.8%, while lipid content decreased significantly by 28.5% compared to their raw equivalents. These modifications are attributed to the metabolic activity during germination, especially the breakdown of macromolecules like carbohydrates into simple sugars that fuel cellular respiration, producing carbon dioxide and water. Additionally, lipid reserves are mobilized as an energy source to support seedling growth [29].

According to a reported protocol, amaranth seeds were steeped at 30 °C for 8 h, then germinated at 23 °C for 24 h, and subsequently kilned at 42 °C for 4 h. This process significantly increased the levels of seven amino acids, particularly aspartic acid, lysine, threonine, leucine, valine, alanine, and serine, while reducing cysteine content, indicating an overall improvement in protein quality. Additionally, the total phenolic compounds, flavonoids, and betalains—especially quercetin, kaempferol, and specific betaxanthins—were elevated. Extended germination and imbibition times further boosted the synthesis of these beneficial compounds [30]. Another study explored the effects of germination temperature and time on the nutrients of amaranth. Grains were germinated for 24–48 h at 22–30 °C. The findings suggest that for amaranth, germination for 48 h at 26 °C was optimal, increasing protein availability by 8%, total energy by 11%, and linoleic acid by 10%, while reducing resistant starch (RS) by 70% [31]. Recently, the effects of germination temperature (24–30 °C) and time (24–72 h) on the nutritional composition and antioxidant properties of amaranth sprouts were evaluated. During germination, sprouts showed a shift from higher hydroxybenzoic acids at 24–48 h to increased hydroxycinnamic acids at 72 h, suggesting changes in phenolic synthesis linked to cell wall formation, particularly hydroxycinnamic acids, which play a crucial role. Gallic and vanillic acids were the most abundant compounds after germination, with concentrations roughly 6.1 and 8.3 times higher than in raw amaranth. The highest levels of bioactive compounds were detected at 30 °C, suggesting that this temperature impacts their accumulation, particularly after 72 h, indicating that it may boost metabolic pathways associated with phenolic synthesis [32]. More recently, germination was reported to occur for 70 h at 23 ± 1 °C under semi-dark conditions, with high relative humidity (~90–95%) maintained by spraying the seeds with water for 15 min every 4 h, followed by drying at 40 °C for 24 h. The present study demonstrates that germination combined with probiotic fermentation enhances the functional and biochemical properties of red and black amaranth seeds. These processes improved acidification potential, antioxidant activity, phenolic and flavonoid profiles, soluble protein content, and probiotic viability in a strain- and variety-dependent manner [5].

A study analyzed by Fractional Factorial Design identified photoperiod as the main factor influencing amaranth seed germination, significantly affecting germination rate and root length, with an 8 h light/16 h dark cycle favoring early seedling growth. Medium composition (Murashige and Skoog without sucrose) was also significant, while hypochlorite washing time impacted germination rate, with 5 min at 3.5% (*v*/*v*) being optimal. Seed density and cold stratification had no significant effects. Significant second-order interactions suggest non-additive behavior. Overall, the light regime is the primary factor influencing early germination in vitro [33].

Various techniques are being explored to enhance seed germination, from traditional priming methods like hydropriming, osmopriming, and hormonal treatments that improve water uptake and enzyme activity [34] to advanced physical treatments such as ultrasound, plasma water, pulsed electric fields, high-pressure processing, and nanopriming with zinc and calcium oxides. Recent innovations include precise physical and nanotechnological techniques like ozone, microwave, UV, and cold plasma treatments, which reduce chemical use. Nanopriming also shows promise under stress conditions like salinity and drought, with methods such as hot air, vacuum drying, and ultrasound accelerating gamma-aminobutyric acid (GABA) accumulation. These approaches, especially when combined with traditional priming, offer sustainable ways to improve germination under challenging environmental conditions [35,36]. For example, a study used a He-Ne laser and magnetic-field pre-sowing stimulation to improve the nutritional profile of amaranth seeds, increasing dry matter, crude protein, crude fibre, and crude ash, while reducing crude oil, carbohydrates, and energetic value. Amino acid levels of leucine, lysine, valine, and phenylalanine plus tyrosine increased, whereas arginine, glutamic acid, and alanine decreased. Magnetic field treatment yielded the most nutritionally effective protein. These non-invasive electromagnetic methods show strong potential for improving amaranth seed nutritional quality, notably by boosting leucine, its typical limiting amino acid [37]. Also, a statistically significant enhancement in germination energy is demonstrated by pre-sowing irradiation of amaranth seeds using either laser light or a magnetic field [38].

Beyond germination, the biological changes initiated in seeds can extend their influence into further processing stages, including fermentation.

## 5. Fermentation of Amaranth Seeds

The rising prevalence of lactose intolerance, *β*-casein allergy, and reduced dairy consumption has driven demand for plant-based milk alternatives, positioning pseudocereals as a highly promising raw material due to their rich nutritional profile and gluten-free nature. However, their antinutritional factors can limit nutrient bioavailability and acceptability, a challenge effectively addressed by lactic acid bacteria fermentation, which reduces antinutrients while generating novel bioactive compounds with antioxidant and probiotic properties [39]. Fermentation improves nutritional value by enhancing protein digestibility, reducing antinutrients such as phytate and tannins, and increasing the bioavailability of essential nutrients, including iron [28].

Plant proteins can be fermented either spontaneously by their native microbiota or through controlled fermentation with selected starter cultures. The choice of microorganisms depends on their functionality, safety, substrate compatibility, and impact on sensory quality. Fermentation efficiency is determined by the interplay between microbial metabolism and the plant matrix’s characteristics, including its carbohydrate content, protein structure, and antinutritional factors [40]. Microbial community analyses have identified several bacterial genera associated with fermented amaranth, including *Pseudomonas*, *Acinetobacter*, *Pectobacterium*, and *Enterobacter* [41]. Also, the bacterial community in fresh amaranth was dominated by *Pantoea agglomerans*, *Pseudomonas oryzihabitans*, and *Cenchrus americanus* [42]. However, the intentional addition of lactic acid bacteria (LAB) can further enhance the fermentation process by promoting more efficient carbohydrate utilization. LAB convert sugars such as glucose and sucrose into organic acids, volatile flavor compounds, and amino acids, thereby improving acidification, flavor development, nutritional quality, and overall functionality of fermented amaranth products. Thus, LAB supplementation represents an effective strategy to optimize fermentation performance and improve the quality and consistency of the final product [43].

Table 2 presents the diverse potential of amaranth species enhanced through fermentation with lactic acid bacteria (LAB), highlighting key effects in both germinated and ungerminated forms of flour.

LAB fermentation combined with germination significantly enhances the nutritional and bioactive qualities of amaranth, making it a valuable ingredient for health-promoting foods. Species such as *Lactiplantibacillus*, *Lacticaseibacillus*, *Leuconostoc*, and *Pediococcus* are commonly used in plant protein fermentation for their robust acidification, substrate adaptability, and safety profile. They enhance protein functionality and digestibility primarily through acidification and proteolysis. Additionally, exopolysaccharide-producing strains improve the texture and mouthfeel of fermented products. Lactic acid bacteria break down antinutrients such as saponins and phytates, improving mineral availability and producing peptides that support blood pressure regulation, immunity, and antioxidant activity [40]. Amaranth fermentation with *Lactobacillus* spp. releases bioactive peptides, including enzyme inhibitors with IC_50_ around 0.47 mg/mL, and enhances antioxidant and antihypertensive profiles, broadening its nutraceutical potential. In amaranth–quinoa matrices, liquid sourdough fermentation achieves nearly 51% protein breakdown and yields ~20.79 g/kg exopolysaccharides, improving texture and bioavailability. Solid-state fermentation, germination–co-fermentation, and fungal fermentation further boost antioxidant capacity, essential amino acids, and phenolics in Andean grains. Among all substrates, amaranth shows the most consistent functional response, highlighting its role in developing next-generation functional foods [44]. Another study shows that the non-volatile metabolite profile of fermented amaranth stems changed markedly during fermentation. Glucose and fructose peaked on day 2, while sucrose declined continuously, indicating microbial sugar utilization. Glycolytic and TCA (tricarboxylic acid) cycle intermediates, along with lactic acid, nucleotides, and several amino acids, generally peaked on day 3, reflecting peak microbial metabolic activity. In contrast, tartaric acid increased continuously throughout fermentation, whereas several amino acids, including glutamic acid, arginine, lysine, and methionine, steadily decreased, likely due to microbial utilization. Overall, the results suggest that the most active metabolic transformations occurred during the first three days of fermentation. Furthermore, a total of 108 volatile organic compounds (VOCs) were identified, and their production was closely associated with active microbial metabolism, with sugar metabolism peaking on the third day of fermentation [41,45]. Phenylpropanoids represent the predominant class of secondary metabolites reported in amaranth and related species, encompassing flavonoids, phenolic acids, and their derivatives, particularly amides. Other major metabolite groups include alkaloids and terpenoids. From a human-centered perspective, compounds such as betacyanins, flavonoids, polyphenols, and phenolic acids are of particular interest due to their contribution to the antioxidant bioactivity of amaranth and quinoa tissues, which underlies many of their recognized health-promoting properties [46,47].

## 6. Amaranth Seed for Next-Generation Derived Products

### 6.1. Applications in Gluten-Free Breads and Bakery Products

Amaranth is an excellent source of high-quality proteins, rich in methionine, cysteine, and lysine. Its protein fraction distribution is more similar to that of pulses than cereals, and it is highly digestible. The main protein components are albumins and globulins, accounting for 50–60% [48]. Furthermore, research investigated phytate breakdown and mineral bioavailability in pseudocereals such as amaranth, fermented either spontaneously or with *Lactobacillus plantarum* 299v. Since phytate hampers the absorption of iron, zinc, and calcium, its reduction is essential to maximize the nutritional benefits of amaranth. Fermentation of amaranth flour was significantly more effective than fermenting whole grains, with phytate reduction mainly due to the grain’s endogenous phytase activity. The addition of *Lactobacillus plantarum* 299v promoted faster pH decline and increased lactic acid production, further improving mineral accessibility by up to 4.6 times. These results demonstrate that fermenting amaranth flour is a practical and efficient method to enhance mineral bioavailability and boost the nutritional value of plant-based diets [49]. It was also found that phytate reduction combined with iron fortification had positive effects on hemoglobin levels and prevented iron deficiency. In children with anemia, those who consumed fermented amaranth bread had higher hemoglobin levels and a lower prevalence of anemia (32% vs. 56%) compared with those who ate control bread [50].

Amaranth combined with rice flour was used to develop a gluten-free steamed bread, with sourdough fermented by *Lactiplantibacillus plantarum* and encapsulated *Limosilactobacillus reuteri* as probiotic strategies. Sourdough fermentation notably improved the amaranth–rice matrix by reducing hardness (~46 N → ~25 N), increasing moisture (38% → ~46%), lowering pH, and enriching the VOC profile with fermentation-derived esters, without altering volume, color, or sensory attributes. Probiotic survival dropped sharply after steaming but partially recovered during storage, while hardness doubled over 10 days—a typical staling behavior in amaranth-based gluten-free breads. Overall, sourdough fermentation proved an effective tool to overcome the textural limitations of amaranth in gluten-free bread formulations [51].

An interesting study made tempeh (a traditional fermented food product originating from Indonesia, obtained by fermenting beans (most often soybeans) with a specific fungus—*Rhizopus oligosporus*) using amaranth seed. Amaranth (*Amaranthus cruentus*) proved to be a promising alternative substrate for tempeh production, owing to its rich nutritional profile—14–18% protein with a complete essential amino acid composition, 14–20% fiber, and meaningful levels of calcium, iron, and B vitamins. At the sensory level, amaranth tempeh stood out for its distinctly clean and neutral profile: consumers scored the amaranth aroma at 3.80/7 and the amaranth flavor at 4.16/7. Its texture was perceived as notably compact (4.88) and rough (4.05), a direct consequence of the grain’s tiny size, which allows hyphae to bind granules into a denser cake [52].

Furthermore, pregnant women consumed amaranth-based flatbread for 6 months, made from 70% amaranth grain (soaked, germinated, roasted, and fermented to improve nutrient bioavailability and reduce phytate levels) and 30% chickpea flour. At the end of the study, the amaranth group had significantly higher hemoglobin levels (12.39 ± 1.34 g/dL) than the maize group (10.17 ± 1.36 g/dL), with an adjusted increase of +2.67 g/dL. The risk of iron deficiency anemia was significantly reduced (ARR = 0.47; 95% CI: 0.26–0.84). Improved hemoglobin, ferritin, and soluble transferrin receptor levels indicated better iron status and bioavailability. The intervention was well accepted and tolerated, supporting the inclusion of nutrient-dense local foods in maternal nutrition programs [53].

Amaranth is a valuable ingredient in gluten-free bread and bakery products due to its high protein and amino acid content, dietary fiber, calcium, iron, and B vitamins, helping to address nutritional gaps. It improves crumb texture, moisture, and shelf life, with 20–40% inclusion recommended for optimal loaf volume and quality [54]. These qualities highlight amaranth’s potential as a health-promoting, functional ingredient in gluten-free baking.

### 6.2. Incorporation into Beverages, Smoothies, and Fermented Drinks

Developing novel functional beverages from blends of cereals and germinated pseudocereals has emerged as a promising strategy to deliver concentrated nutritional and bioactive benefits in a convenient, palatable form while addressing current nutritional challenges [55]. Among the pseudocereals explored, amaranth (*Amaranthus* spp.) stands out for its exceptional nutritional profile. Germination further enhances these attributes by stimulating the synthesis of phytochemicals linked to improved oxidative stress management and disease prevention. In the study, amaranth grains were germinated at 30 °C for 96 h, then dried and ground into coarse flour. The resulting functional drink—formulated at a malt-drink-to-milk ratio of 40: 20: 40 (buckwheat: amaranth: oats)—showed notably high levels of dietary fiber (2.93%), *β*-glucan (0.89%), total polyphenols (248.84 mg GAE/100 g), and antioxidant activity (DPPH: 30.65%; ABTS: 19.63%), along with an exceptional calcium content of 1452.6 mg/L and a low fat content (1.23%), largely attributable to amaranth’s inherent mineral richness and bioactive density [56].

Beyond germinated grain beverages, amaranth proteins themselves have shown strong potential as the primary structural and nutritional basis for a novel plant-based drink. Amaranth proteins have also proven effective as the sole structural basis for a stable, nutritious plant-based beverage. By combining amaranth protein extract with gellan and xanthan gums, followed by heat treatment at 80 °C for 20 min, a beverage compositionally comparable to skim cow’s milk was obtained (protein: 3.4%, lipids: 0.6%, fiber: 1.9%, carbohydrates: 3.0%, water: 90.6%). The gums attenuated thermal denaturation of the proteins and promoted the formation of gum–protein macrocomplexes with enhanced ζ-potential, ensuring high colloidal stability—as confirmed by electrophoresis, DSC, and particle size analysis. The resulting product is gluten-free, lactose-free, and suitable for vegans, reinforcing amaranth’s versatility as a key functional ingredient in the development of inclusive, health-promoting beverages [57].

Further, a study developed functional beverages from amaranth flour (processed by germination and extrusion) blended with germinated chia flour and evaluated their nutritional, antioxidant, and antihypertensive properties. Optimal processing conditions were applied to extruded amaranth flour (EAF: 141 °C/81 rpm) and germinated amaranth flour (GAF: 30 °C/78 h), alongside germinated chia flour (GCF: 29 °C/197 h), to enhance bioactive compounds. Formulations with 70% EAF and 30% GCF, or 70% GAF and 30% GCF, provided 3.90–4.53 g protein, 5.04–6.81 g dietary fiber, and 95–96 kcal, with a protein efficiency ratio of 2.52–2.69. The beverages showed high antioxidant activity (4.009–6.495 µmol TE) and strong antihypertensive potential (IC_50_ = 0.43–0.47 µg extract/mL). Sensory acceptability ranged from “I like it very much” to “I like it extremely,” indicating strong consumer acceptance. Overall, amaranth (at 70% inclusion) was the main contributor to the functional properties, confirming its potential as a key ingredient in nutrient-dense, antioxidant-rich functional beverages [29]. Moreover, a patent specifies the method for producing vegetable milk from amaranth seeds. It involves sprouting the seeds in electrophysically activated water with a low, constant electric current (0.001–1 mA) until the sprouts reach 1–2 mm in length, followed by mineralization and recirculating filtration assisted by underwater ultrasound (22 kHz frequency, up to 70 μm vibration amplitude, and 0.1–0.6 kW/cm^2^ power density). After this pretreatment, the seeds are ground into a homogeneous mass, extracted in hot water at a solid-to-liquid ratio of 1:3–1:5, and finally subjected to filter pressing. This method boosts soluble protein and enhances digestibility in vegetable milk. It confirms amaranth as a key ingredient to improve the nutritional and health benefits of plant-based drinks, which can be consumed directly, pasteurized, stored for 48 h, or used in other food products [58].

### 6.3. Use in Nutraceuticals and Functional Powders

Amaranth seeds are increasingly used as a healthy seasoning and supplement in porridges, baked goods, pasta, and confections because they surpass most cereals in protein, oil, fiber, and lysine content. For instance, 100 g of amaranth protein provides 6.2 g of lysine, an essential amino acid scarce in other plants. Lack of lysine impairs nutrient absorption and causes proteins to pass through the body unused.

Furthermore, amaranth grain is remarkably high in soluble dietary fiber, which has a strong prebiotic effect in the gut by encouraging the growth of protective microbes and boosting their production of short-chain fatty acids (SCFAs). These SCFAs are crucial for maintaining intestinal balance and overall health. Among pseudocereals, amaranth and its grain components, such as proteins and polysaccharides, show promising potential to influence the composition of the gut microbiota. The bioactive compounds metabolized by these microorganisms also have positive effects on host metabolism. Furthermore, dietary supplementation with amaranth grain fractions increases SCFA levels, boosts gut microbiota diversity, and helps prevent dysbiosis caused by a high-fat diet [59].

Key values of amaranth for use in food supplements include its status as a rich source of SCFA and polyphenolic compounds, found in seeds and oil, which are used in food matrices and food supplements (nutraceuticals) [60]. Inclusion in functional powders aims to improve overall protein quality. Adequate dietary protein is crucial, as protein is the main structural component of body cells and is essential for maintaining cellular integrity, function, health, and reproduction. Vegetable proteins are valuable due to their high biocompatibility, nutritional value, and low cost, especially in foods rich in essential amino acids. Regarding amaranth, its protein quality parameters include a digestibility of 79.25%, a chemical score of 47.59%, an essential amino acid index of 59.76%, and a protein efficiency ratio of 6.17 [61].

### 6.4. Integration into Feed and Animal Nutrition

Amaranth is an underutilized and neglected crop whose grains and leaves are consumed by both humans and animals. Amaranth is a promising alternative crop for food security because of its flexibility, drought- and heat-resilience (ability to adapt to different soil and climate conditions), and high yield potential. Also, replacing amaranth at relatively low concentrations has no adverse effects on animal feeding [62]. Moreover, amaranth’s high protein content is a valuable ingredient in animal feed, particularly for livestock that needs extra protein for growth and muscle development and improving digestibility. Adding amaranth powder to feed mixes may improve animal performance. Amaranth leaves and grain are a potential source of methionine and protein for monogastric animals. The goal of monogastric nutrition is to provide the optimal quality and quantity of nutrients to meet all animals’ needs for growth, egg production, and maintenance [63]. Furthermore, in feed formulation, germination can improve protein digestibility by breaking down antinutritional factors and activating enzymes (such as phytase, amylase, and protease) that cleave complex molecules into smaller components. After 72 h of germination, amaranth seed flour shows reductions of 75.7% in tannins, 62.3% in oxalates, 65.7% in phytates, and 32.0% in trypsin [64]. A study on germinated amaranth consumption in mice showed that germination enhances the nutritional value and biofunctional properties of amaranth seeds by increasing the availability of bioactive compounds. Supplementation of animal feed with amaranth seeds or sprouts for 28 days reduced body weight and improved metabolic parameters, including total cholesterol, LDL cholesterol, atherogenic index, and blood glucose, under both balanced and hypercaloric diets. These beneficial effects are attributed to bioactive constituents such as peptides, fatty acids, squalene, and phenolic compounds. Although these findings highlight the potential of germinated amaranth to improve lipid and glucose metabolism, clinical studies are needed to confirm its efficacy in humans [47]. Another interesting study revealed that a feeding trial with laying hens showed that dietary raw amaranth grain (RAG) improved blood lipid profiles, antioxidant status, and egg quality. This included reductions in total cholesterol, the atherogenic index, and egg yolk cholesterol. However, incorporating RAG at levels above 10% negatively affected production performance, including feed efficiency, egg weight, and egg mass. The addition of an enzyme blend mitigated these adverse effects, enabling the inclusion of up to 10% RAG without harming productivity. These benefits are attributed to the high levels of bioactive compounds in amaranth, including phytosterols, tocopherols, and squalene, emphasizing its potential as a functional feed component for producing healthier, low-cholesterol eggs while supporting poultry performance [65]. Moreover, starchy feeds such as cereals, legumes, and potato starch serve as primary energy sources in pig diets. Dietary lipids can affect nutrient absorption and gut fermentation. Unsaturated long-chain fatty acids, especially n-3 PUFAs, may enhance lipid digestion and influence the gut microbiota by altering bacterial populations and increasing short-chain fatty acids (SCFAs). For instance, amaranth varieties with higher oleic acid, α-linolenic acid, MUFA, and n-3 PUFA levels showed better organic matter digestibility, fermentation, and SCFA production. Conversely, linoleic acid and n-6 PUFA were less beneficial or negatively associated. Among these, *A. hypochondriacus*, with elevated α-linolenic acid and n-3 PUFA, demonstrated superior digestibility, fermentation, and SCFA outputs—particularly propionate and butyrate. These results suggest that amaranth’s lipid composition could enhance gut microbial activity, supporting energy metabolism and intestinal health in animals [66]. Dietary supplementation with up to 20% heat-treated *Amaranthus spinosus* was found to reduce abdominal fat in a linear pattern (*p* = 0.032) and to enhance meat antioxidant status in male Ross 308 broiler chicks [67]. Evidence indicates that appropriate inclusion levels support animal performance without adverse effects, with up to 40% fresh or ensiled amaranth suitable for ruminants, up to 20% heat-treated grain for poultry, and up to 10% amaranth leaf meal for pigs. Overall, amaranth has considerable potential to improve the sustainability and cost-effectiveness of livestock production [62].

## 7. Conclusions

An innovative concept involves developing functional ingredients by combining amaranth seed germination and fermentation. This concept proposes the strategic integration of germination and fermentation as complementary bioprocessing techniques to produce high-value functional ingredients from amaranth seeds. Germination activates endogenous enzymatic systems—including proteases, amylases, and phytases—initiating the biochemical transformation of seed constituents and enhancing the overall nutritional profile. When coupled with controlled fermentation processes—whether lactic acid, mixed-culture, or starter culture-driven—this dual bioprocessing approach further modulates the matrix’s chemical composition, yielding products with superior nutrient bioavailability, enriched bioactive compound content, and improved techno-functional properties relevant to food manufacturing. Despite its considerable potential, this integrated approach remains insufficiently explored in the scientific literature. Key knowledge gaps persist regarding the microbiological and biochemical dynamics governing the combined process, the systematic optimization of technological parameters (time, temperature, inoculum type and concentration, substrate-to-water ratio, etc.), and the comprehensive evaluation of the functional and health-promoting attributes of the resulting ingredients. Addressing these gaps represents a scientifically relevant and practically significant research direction. This concept opens up new avenues for the design of innovative, nutritionally dense, and sustainably produced food products with demonstrable health benefits, with strong applicability across the food industry, nutraceutical sector, and functional ingredient market.

## Figures and Tables

**Table 1:** Nutritional composition of amaranth (*Amaranthus* spp.) seeds.

Nutrient/Component	Content	Reference
Protein	12.0–18.3%	[14,15]
Fat	2.23–4.83%	
Starch	49.5–63.0%	
Sugars	2.0–3%	[16,17]
Dietary fiber	8.0–15%	
Oil	7.0–9%	
Lipids	6.98–7.22%	[18,19]
Oleic and linoleic acids	35–70%	
Stearic acid	4–20%	
Linolenic acid	1–2%	
Phosphorus	558.1–738 mg/100 g	[16,20,21]
Potassium	337–509.1 mg/100 g	
Magnesium	218–249.1 mg/100 g	
Calcium	158.5–223 mg/100 g	
Iron	7.7–8.3 mg/100 g	
Sodium	4.1–6.30 mg/100 g	
Manganese	3.3–4.54 mg/100 g	
Zinc	2.9 mg/100 g	
Copper	0.6–1.03 mg/100 g	
Selenium	18.76 µg	
Vitamin B9	82.2 mg	
Vitamin B12	69.8 mg	
Vitamin C	4.2 mg	
Vitamin B5	1.5 mg	
Vitamin B3 (niacin)	0.9–1.02 mg/100 g	
Vitamin B2 (riboflavin)	0.131–0.2 mg/100 g	

**Table 2:** Enhancing amaranth’s potential through fermentation processes.

Seed Species	Seed Germination Status	LAB	Effects	Ref.
*Amaranthus* *hypochondriacus*	ungerminated	*Lactiplatibacillus plantarum*, *Enterococcus* spp., *Leuconostoc* *mesenteroides*	Effectively degrades proteins to produce bioactive hydrolysates with antioxidant, antihypertensive, and antimicrobial properties, as well as multifunctional applications in functional foods and nutraceuticals.	[11]
*A.hypochondriacus*/*A. cruentus* and *A. caudatus*	germinated	*Lacticaseibacillus rhamnosus*	Enhancing acidification, boosts soluble protein release, improves antioxidant potential, and maintains probiotic viability; especially red amaranth is a promising gluten-free ingredient for innovative probiotic and nutraceutical food formulations.	[12]
*A. caudatus*	ungerminated	*L. plantarum*	Fermenting pseudocereal flours significantly improves mineral accessibility and bioavailability. This enhancement results from phytate breakdown and the preservation of mineral content during processing. Incorporating fermented pseudocereal flour can enhance the nutritional quality of plant-based diets.	[13]
*Amaranthus* spp.	ungerminated	*L. plantarum*	The production of short fermented (15 h) liquid sourdoughs, enriched in EPS, for bread-making.	[14]
*A. caudatus* var. Alegrìa	germinated	*L. plantarum*	Germination increased macro- and microelements, bioactive compounds, and antioxidant properties. Fermentation further boosts antioxidant content, enhancing the potential benefits of the fermented product.	[15]
*Amaranthus* spp.	ungerminated	*Enterococcus* *faecium*	Production of protein hydrolysates with peptides showing potential ACE-I inhibitory activity; 125 peptides with unreported ACE-I activity were identified. Peptides IFQFPKTY and VIKPPSRAW strongly interacted with ACE by binding to its active site.	[16]

## Data Availability

The original contributions presented in this study are included in the article. Further inquiries can be directed to the corresponding author.
